# Editorial: Responding to the COVID-19 Pandemic: Health Technology Solutions to Improve Access and Delivery of Cognitive Behavior Therapy

**DOI:** 10.3389/fpsyg.2022.901399

**Published:** 2022-04-12

**Authors:** Brian E. Bunnell, Judith A. Callan, Nikolaos Kazantzis

**Affiliations:** ^1^Department of Psychiatry and Behavioral Neurosciences, Morsani College of Medicine, University of South Florida, Tampa, FL, United States; ^2^Doxy.me Research, Doxy.me Inc., Rochester, NY, United States; ^3^School of Nursing, University of Pittsburgh, Pittsburgh, PA, United States; ^4^VA Pittsburgh Health Care, MIRECC, Pittsburgh, PA, United States; ^5^Cognitive Behavior Therapy Research Unit, Melbourne, VIC, Australia; ^6^Beck Institute for Cognitive Behavior Therapy, Philadelphia, PA, United States

**Keywords:** COVID-19 pandemic, health technology, mental health, cognitive behavior therapy (CBT), telemedicine

## Introduction

The COVID-19 pandemic has substantially impacted physical and mental health for the international community (Ceban et al., 2021). While initial reports suggest substantial psychological impact of contracting COVID-19, or fearing infection, or witnessing a close family member struggle with vaccination or treatment of the virus (Weiner et al., 2021), it is clear that social distancing and stay-at-home guidelines have had their own effects (Knights et al., 2021). There has been an unprecedented need for solutions to support public mental health and mental health providers in delivering remote patient care.

Fortunately, the advances of technology platforms for telehealth and the delivery of evidence-based psychological therapies have progressed to a point where evidence for their effectiveness is well known and the ubiquity of smartphone and other mobile technology resources has helped our international communities during these difficult times (e.g., Bakker et al., 2018; Callan et al., 2021). As such, we sought to produce this Research Topic for *Frontiers in Psychology* to showcase some of the important work being done to improve access and delivery of Cognitive Behavior Therapy (CBT).

## Responding to the COVID-19 Pandemic

The pandemic led to a rapid transition to the remote delivery of mental health care. Some studies found that over 97% of mental health providers began using remote-based care to supplement or replace in-person care, and that most of these providers plan on continuing its use following the pandemic (Douglas et al., 2017; Slone et al., 2021; Zhu et al., 2021). Additionally, digital interventions such as eHealth and mHealth are considered to have been integral in supporting mental health and its care during the pandemic (Rauschenberg et al., 2021; Wang et al., 2021). It is therefore imperative to better understand these solutions and their use to inform and sustain post-pandemic technology-augmented healthcare models.

Our aim in producing this Research Topic was to bring together research studies on health technology solutions to improve access and delivery of CBT, with a particular focus on their potential impact relative to COVID-19. We were interested in articles on innovative health technology solutions that support, augment, or independently deliver CBT (e.g., telemedicine, eHealth, mHealth), and how they could be used to mitigate the negative mental health outcomes of the pandemic. The goal was to provide a stronger evidence-base for these health technology solutions and support providers in their use during and after the COVID-19 pandemic.

## Articles in This Research Topic

Arjmand et al. present data (*N* = 755) from an Australian study that used a smartphone-based mood monitoring application to measure changes in mental health and well-being in response to the Australian bushfires and the pandemic. Results showed that while anxiety symptoms did not increase in response to the bushfires, anxiety and depressive symptoms increased significantly during the pandemic. This work demonstrates the potential utility of technology for informing public health interventions as well as individual clinical treatment in the context of environmental disasters Oliveira et al. conducted a systematic review exploring mHealth intervention conceptual frameworks, acceptability, and efficacy relative to mental health in college students during the pandemic. The results suggested that college students accept and adhere to mHealth interventions. Further preliminary evidence for the efficacy of CBT-based mhealth interventions for college students was found for stress, anxiety, depression, and risky behaviors. This work highlights how mhealth can promote and deliver CBT-based interventions at a safe distance, which is particularly important in the context of a global pandemic.

Soares Ribeiro et al. examined the psychometric properties of a cross-cultural adaptation of the Working Alliance Inventory-Short Form-Observer (WAI-SR-O) with a Brazilian sample (*N* = 10) during telehealth-based CBT for alcohol addiction. The results provided preliminary support for the reliability and validity of the WAI-SR-O in this sample and treatment context but do require further investigation with a larger sample. Working alliance is considered an evidence-based relational process in CBT (Del Re et al., 2021), and it is important to examine whether meaningful assessments are possible when such measures are translated, but even more crucially for the present context, when CBT is delivered by telehealth.

Bunnell et al. conducted a survey and comparison of CBT vs. non-CBT telemental health providers in the USA (*N* = 276). Findings indicated that CBT-based telemental health providers were more likely to use evidence-based practices such as introducing and practicing therapeutic exercises (e.g., behavioral activation, exposure) in-session, assigning patients to practice those exercises between-session (i.e., for homework), and collecting clinical data from their patients. However, most telemental health providers reported doing so verbally, mail, or email, methods that can be less effective, efficient, and secure. The authors highlighted the need for innovative solutions to improve these processes for telemental health providers.

Aminoff et al. presented data from a pilot randomized controlled trial in Sweden where patients (N = 52) were randomly allocated to a brief, 7-week individually tailored Internet-based CBT (ICBT) or wait list control. The preliminary findings were supportive and suggest that relatively brief ICBT with some amount of tailoring to the specific individuals' needs can reduce pandemic-related stress, depression, and anxiety symptomatology. These findings are encouraging given ICBT's potential to increase access to CBT on a widespread, and international level.

Finally, Zhao et al. examined psychological risk factors for “untact” buying behavior (i.e., buying with no human contact) among a large group of Chinese university students (*N* = 1,564). The study examined the well-known psychological construct of “intolerance for uncertainty” and examined the mediating role of perceived risk of COVID-19 and “protection motivation” in explaining buying behaviors. These findings have important clinical and public health implications.

## Conclusion

This Research Topic for *Frontiers in Psychology* provides a rich sample of contemporary work focused on advancing our understanding of how technology can improve access and delivery of assessment and interventions based within CBT. We were fortunate to have such participation from researchers from multiple continents, especially given the worldwide effects of the pandemic. We hope these studies will inspire continued research on health technology solutions to measure the impact of global disasters, solicit feedback from health professionals, objectively assess how technology may facilitate or hinder the delivery of CBT, and of course, encourage new technology innovations for tailored mental health care.

## Author Contributions

All authors contributed to the article and approved the submitted version.

## Funding

BB was funded by the National Institute of Mental Health (Grant Numbers K23MH118482 and R41MH126734).

## Conflict of Interest

BB was an employee of Doxy.me, Inc., a Commercial Telemedicine Company and also CEO of Adhere.ly, LLC, a Mental Health Technology Company. The remaining authors declare that the research was conducted in the absence of any commercial or financial relationships that could be construed as a potential conflict of interest.

## Publisher's Note

All claims expressed in this article are solely those of the authors and do not necessarily represent those of their affiliated organizations, or those of the publisher, the editors and the reviewers. Any product that may be evaluated in this article, or claim that may be made by its manufacturer, is not guaranteed or endorsed by the publisher.
